# Development of Multiple Myeloma of the IgA Type in a Patient with Cold Agglutinin Disease: Transformation or Coincidence?

**DOI:** 10.1155/2019/1610632

**Published:** 2019-03-04

**Authors:** Øystein Sefland, Ulla Randen, Sigbjørn Berentsen

**Affiliations:** ^1^Section of Hematology, Department of Medicine, Haukeland University Hospital, Bergen, Norway; ^2^Department of Pathology, Akershus University Hospital, Lørenskog, Norway; ^3^Department of Research and Innovation, Haugesund Hospital, Haugesund, Norway

## Abstract

Cold agglutinin disease (CAD) is an autoimmune hemolytic anemia and a distinct, clonal bone marrow lymphoproliferative disorder, characterized in most cases by a monoclonal IgMκ serum protein. We describe a CAD patient presenting with a monoclonal immunoglobulin of the IgA*λ* class. For years, she remained asymptomatic apart from the hemolytic anemia until eventually she developed multiple myeloma (MM) of the IgA*λ* phenotype. Prior to the development of MM, her hemolytic anemia responded to rituximab monotherapy. After she was diagnosed with MM, both conditions responded well to bortezomib-based therapy. We performed further investigations to determine whether her MM represented a progression/transformation of CAD or an independent disease. Flow cytometry and biopsy findings convincingly confirmed two unrelated clonal B-cell disorders. On this background, we also discuss previously published reports on cold agglutinin activity in patients with IgA gammopathy. In conclusion, cold agglutinins of the IgA class do probably not result in CAD. If a monoclonal immunoglobulin other than IgMκ is found in a patient with CAD, the coexistence of two unrelated B-cell clones should be suspected.

## 1. Introduction

Primary chronic cold agglutinin disease (CAD) is an uncommon type of autoimmune hemolytic anemia (AIHA) and is today considered a specific clonal B-cell lymphoproliferative disorder (LPD) of the bone marrow [1–4]. The involved autoantibodies, known as cold agglutinins (CAs), are monoclonal and directed against the erythrocyte surface carbohydrate antigen termed I [2, 5]. CAs in CAD are almost invariably of the immunoglobulin (Ig) Mk class, whereas descriptions of IgG or IgA immune phenotypes are rare, as it is *λ* light chain restriction [6–9]. CAD mainly affects elderly or middle-aged people with a median age of 72 years [7].

The underlying bone marrow lymphoproliferation, when demonstrable, is clearly indolent by histology and can hardly be considered a malignant disease in a clinical sense. Median survival has been estimated to be 12.5 years from diagnosis, which does probably not differ much from the general expected survival in this elderly population [7]. Transformation to aggressive lymphoma is an uncommon event, shown to occur in only 3-4% of the patients during a median observation period of more than 10 years [7]. Transformation to, or coexistence with, multiple myeloma (MM) has only been reported in a couple of cases worldwide [9].

Herein, we describe a patient with a stable CAD and an IgA*λ* monoclonal spike on serum electrophoresis, who for years remained asymptomatic apart from the hemolytic anemia and then developed MM of the IgA*λ* phenotype. We consider this report highly relevant for colleagues caring for patients with CAD, in particular those who might encounter patients with CAD of apparent non-IgM phenotype.

## 2. Case Presentation

A woman in her late 60s was transferred in July 2012 from her local hospital because of anemia of at least one and a half year duration. She had no relevant family history, but had been treated for diabetes type 2 and hypertension for decades and paroxysmal atrial fibrillation for a few years. She had suffered from recurrent urinary tract infections, several times with fever and once with verified urosepsis. A concrement had been removed from her right ureter a few months ago.

Her history of anemia started gradually with fatigue in 2010-11. Her hemoglobin (Hb) level was 8.9 g/dL in February 2011, as compared to 13.4 at the last known previous assessment in 2008. She had received an erythrocyte transfusion at her local hospital without any transfusion reaction or other problems, and she had already suffered several exacerbations of anemia during febrile infections. There was no history of acrocyanosis or Raynaud phenomena.

On admission, she was in good general condition and did not present any pathological findings by physical examination. In particular, there was no acrocyanosis, lymphadenopathy, or splenomegaly. Chest radiography and abdominal ultrasonography were unremarkable. Hb was 8.2 g/dL, leukocytes 7.8 × 10^9^/L with normal differential count, platelets 263 × 10^9^/L, mean corpuscular volume (MCV) 99 fL, reticulocytes 88 × 10^9^/L, and C-reactive protein (CRP) 11 mg/L. Serum levels of iron, transferrin, cobalamin, and folic acid as well as transferrin saturation were within the reference range, however, with elevated ferritin at 1257 *μ*g/L. Genetic screening for hemochromatosis showed no HFE gene abnormalities. Concentrations of electrolytes, calcium, creatinine, and liver transaminases were normal. Lactate dehydrogenase (LDH) was elevated at 544 U/L, bilirubin elevated at 43 *μ*mol/L, while haptoglobin was undetectable (less than 0.1 g/L). Her urine was negative for hemoglobin and protein. Serum albumin was 41 g/L with normal immunoglobulin levels (IgG 7.0 g/L, IgA 3.2 g/L, and IgM 0.69 g/L). Serum electrophoresis identified a small spike of monoclonal IgA*λ*. Free *λ* chains in serum were slightly elevated at 53 mg/L, however, with a *κ*/*λ* ratio within the reference range. The direct antiglobulin test (DAT) was strongly positive for C3d and negative for IgG, IgM, and IgA. CA titer at 4°C was 128. Serum erythropoietin was slightly elevated at 36 IU/L.

Based on these findings, she was diagnosed with CA-mediated AIHA. With a chronic course, no signs of malignancy so far, and no recent specific infection, this AIHA was further classified as primary CAD. A bone marrow trephine biopsy showed erythroid hyperplasia and small lymphocytic infiltrates interpreted as lymphoplasmacytic lymphoma (LPL). Flow cytometry in bone marrow aspirate revealed two small, clonal populations: one of B-lymphocytes that displayed a *κ* phenotype and the other one of *λ* positive plasma cells.

**Figure 1:**
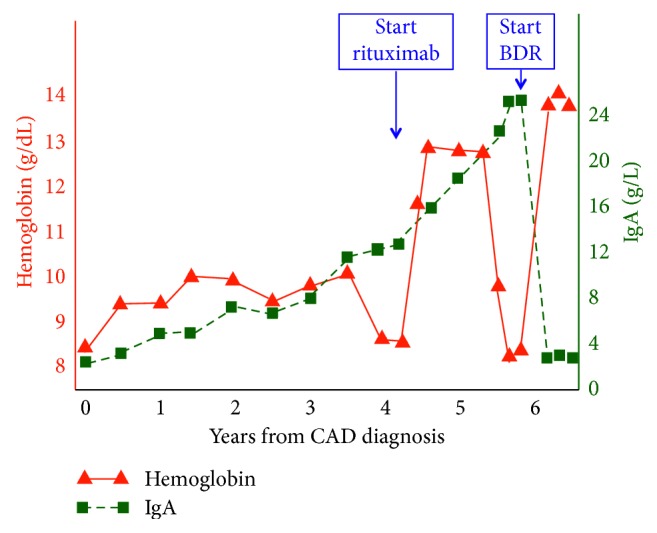
Changes in hemoglobin and IgA levels. BDR: bortezomib, dexamethasone, and rituximab combination therapy. CAD: cold agglutinin disease.

The further development in Hb and IgA levels is shown in Figure 1. During the next couple of years, Hb ranged from 9.0 to 10.0 g/dL; she had only mild fatigue and no transfusion requirement. Management consisted of regular follow-up, avoidance of cold exposure, and prompt antibiotic therapy in case of febrile bacterial infection but no CAD-directed pharmacological therapy. By 2014, a second bone marrow biopsy showed erythroid hyperplasia and lymphoid infiltrates, now interpreted as probable CAD-associated LPD [4]. Flow cytometry in bone marrow aspirate again demonstrated 2 pathological clones of *κ* positive B-lymphocytes (approximately 5%) and *λ* positive plasma cells (0.7%), respectively.

Her condition remained stable until late spring 2016, when she deteriorated. Her fatigue worsened, her atrial fibrillation relapsed more frequently, and her Hb level declined to 8.5 g/dL. The reticulocyte count was now elevated at 191 × 10^9^/L, LDH was 303 U/L, bilirubin 96 *μ*mol/L, haptoglobin <0.1 g/L, while IgA had increased to 12.5 g/L and CA titer to 512. The DAT findings were unchanged. We decided to give her rituximab monotherapy [10], four weekly doses of 375 mg/m^2^, which she received from August 2016. Hb rose to 12.5 g/dL by December 2016, while bilirubin and LDH normalized. Unexpectedly, however, her IgA level continued to increase from 12.5 g/L immediately before rituximab treatment to 19.6 g/L by January 2017.

From June 2017, Hb started again to decline and IgA continued to increase. In January 2018, she experienced severe lumbar pain after having removed snow from her courtyard, which can be heavy work in Norway. One month later, Hb was 9.5 g/dL, while the absolute reticulocyte count was 153 × 10^9^/L and the levels of LDH, bilirubin, and haptoglobin had not changed significantly. The IgA level was 24.5 g/L and *β*2-microglobulin 3.5 mg/L. A new trephine biopsy showed hypercellular bone marrow affected by 20% infiltration of monoclonal, *λ* positive plasma cells, and some small lymphocytic infiltrates. A CT scan showed osteolytic lesions of the skull but no relevant pathological findings in her spine, pelvis, or femora. She was diagnosed with development of MM.

In February 2018, a local visiting hematologist initiated a cycle of melphalan and prednisolone. During the next couple of weeks, Hb declined further to 8.0 g/dL, however, without any neutropenia or thrombocytopenia. Concomitantly, a rise in bilirubin and LDH was observed, and she was diagnosed with an exacerbation of CAD. Melphalan and prednisolone were discontinued after the first cycle.

Hoping to induce remission of both MM and CAD, we decided in March 2018 to offer her bortezomib-based therapy [11] with the addition of dexamethasone and rituximab [10, 12, 13]. She received four cycles at four weeks' interval of bortezomib (1.3 mg/m^2^ weekly for 3 weeks), rituximab (375 mg/m^2^ once per treatment cycle), and dexamethasone (20 mg on days 1, 2, 8, 9, 15, and 16). She tolerated this combination well; her Hb level increased from 8.0 g/dL to 13.6 g/dL following the first cycle and reached 14.4 g/dL after the second one. Bilirubin and LDH normalized within a couple of weeks, and reticulocyte count declined to the normal range (65 × 10^9^/L). IgA had declined to 3.8 g/L by the start of the second cycle. Her back pain improved. Ten weeks after start of therapy, Hb was 13.2 g/L, bilirubin 7 *μ*mol/L, LDH 247 U/L, haptoglobin 0.24 g/L, and CA titer 64. IgA remained in plateau at 3.9 g/L, while serum electrophoresis did not show any monoclonal spike. DAT was still positive for C3d.

### 2.1. Bone Marrow Biopsy Data, Revised

**Figure 2:**
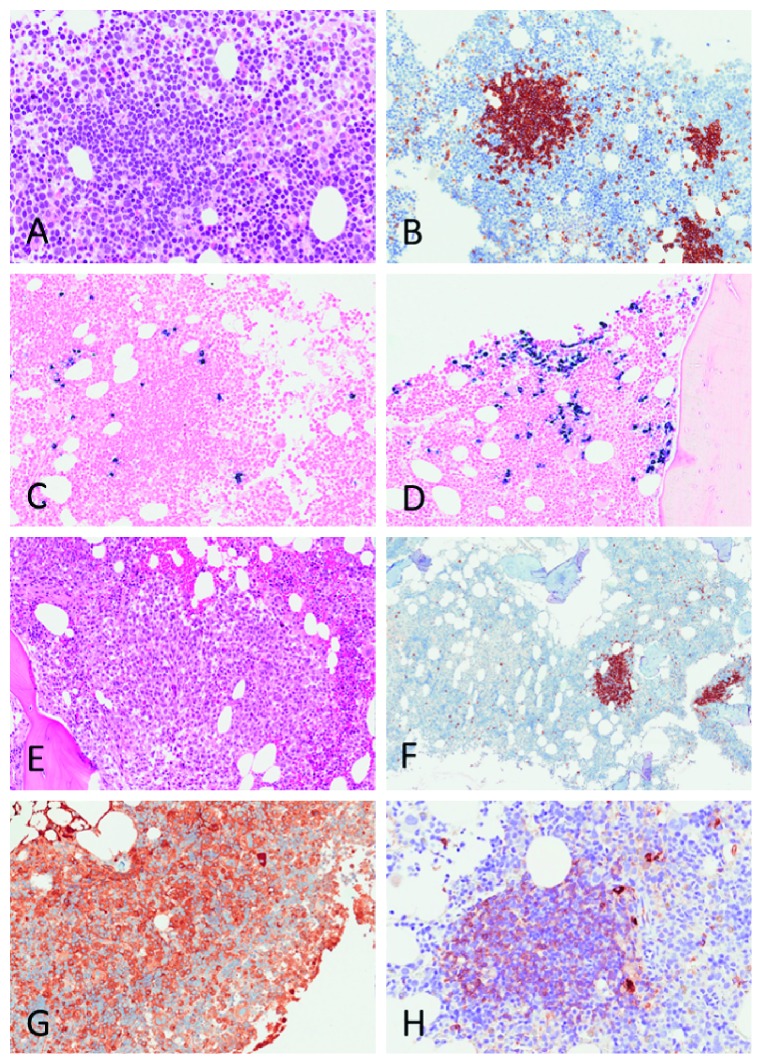
Bone marrow trephine biopsies from 2012 (a–d) and 2018 (e–h), respectively. The nodular lymphoid infiltrates (a) are CD20 positive (b) and surrounded by *κ* positive plasma cells (c), typical for CAD. In other areas, there are small focal aggregates of *λ* positive plasma cells (d), consistent with MGUS. In the 2018 biopsy, there is extensive plasma cell infiltration (e), consistent with MM. CD20 staining (f) shows remaining CD20 positive CAD infiltrates. The MM is IgA positive (g), whereas the CAD infiltrates are IgM positive (h).

The lymphoma pathologist in our group (U.R.) did a centralized, parallel review of all three bone marrow samples based on extensive experience with CAD-associated bone marrow pathology [4]. The 2012 biopsy shows, in addition to erythroid hyperplasia, intraparenchymatous, *κ* positive B-cell infiltrates consistent with CAD-associated LPD. There are scattered *κ* positive plasma cells, more of them near the lymphocytic infiltrates, and one distinct infiltrate of *λ* positive plasma cells. In the 2014 biopsy, the picture has not changed significantly. The trephine biopsy from February 2018 shows an IgA*λ* positive plasma cell population that has expanded, but IgMκ positive B-cell infiltrates can still be seen. In retrospect, therefore, all three biopsies show two clones; and in the 2018 sample, the CAD-associated IgMκ plasma cell population seems “hidden” among the expanding, myeloma-associated IgA*λ* plasma cells (Figure 2).

## 3. Discussion

The patient history raises the question of whether she suffered a transformation of CAD-associated low-grade LPD to an overt B-cell malignancy or had two independent B-cell proliferative diseases. This issue also relates to previously published reports on IgA-mediated CAD.

CAD has been shown by our group and others to be a distinct, low-grade bone marrow LPD, typically different from LPL, marginal zone lymphoma, and other mature B-cell LPDs [4, 14, 15]. An underlying mutated B-cell clone seems present even in patients in whom no LPD or gammopathy is detected by electrophoresis, flow cytometry, or histology [16]. Transformation to aggressive B-cell lymphoma is a rare event, probably occurring in less than 4% of the patients during an observation period of more than 10 years [7]. In the vast majority, a monoclonal serum immunoglobulin can be demonstrated if sensitive techniques are used, most often of the IgMκ class [7, 17, 18]. However, IgA-CAs have been reported in a few cases [8].

Plasma cell neoplasms are classified into three stages: monoclonal gammopathy of undetermined significance (MGUS), smoldering myeloma (SMM), and multiple myeloma (MM), of which the last two are defined as malignant diseases and the MM stage is an indication for therapy [19–21]. With serum monoclonal IgA > 20 g/L, 20% clonal plasma cell infiltration in the bone marrow, skeletal pain, and several cranial osteolytic lesions, our patient had reached the MM stage by the beginning of 2018. MM and SMM are assumed to be preceded by MGUS in all cases [19]. The rate of progression to MM in individuals with IgG- or IgA-MGUS has been estimated to approximately 1% per year, with variations depending on risk factors [20]. MM is of the IgA subtype in approximately 25% of the patients [21].

In our patient, therefore, it was tempting to assume a progression or transformation of a low-grade LPD in CAD, also manifested by a monoclonal IgA gammopathy, to an established B-cell malignancy presenting as MM. Already in 2012, however, there was a discrepancy between the demonstration of a cell clone expressing the *λ* light chain phenotype, as shown by electrophoresis and histology, and the additional finding of *κ* positive clonal cells. In retrospect, all three bone marrow examinations provide evidence of two different B-cell clones, one compatible with gammopathy of the IgA*λ* class, starting with MGUS and progressing to MM, and the other one of the IgMκ phenotype and typical for CAD-associated LPD. The latter did not manifest as a detectable monoclonal gammopathy at any time. For the same reasons, it is highly unlikely that both clones could have evolved from a common early progenitor B-cell.

In patients with CAD responding to B-cell directed therapies, the clinical response with normalization of Hb and parameters of hemolysis is associated with a decrease in the involved monoclonal immunoglobulin and a regression of any morphological signs of LPD [10, 13]. In our patient, however, IgA continued to increase in parallel with the improvement of CAD following rituximab monotherapy. This fits well with the demonstration of two independent clones. The non-IgA nature of the CA in our patient might have been further confirmed by agglutination-elution techniques and electrophoresis of purified CA [17]. In theory, it might also have been possible to further confirm two different clones by molecular studies of cells sorted by flow cytometry-assisted cell sorting [15]. Currently, after achievement of remission with no detectable monoclonal protein and low CA titer, neither of these approaches will be feasible.

The reticulocyte count is usually, but not invariably, increased in patients with AIHA [18]. Based on the normal reticulocyte count at diagnosis, one might speculate whether the coexisting plasma cell clone inhibited the reticulocyte response in some way. However, the later development of reticulocytosis in parallel with the progression of the plasma cell clone excludes this possibility. The reticulocyte count normalized again after achievement of remission with abrogation of hemolysis.

Six case observations have previously been published describing IgA gammopathy in association with the occurrence of CA in serum. The three oldest reports described monoclonal CAs of the IgA class without hemolysis, that is, these individuals did not have CAD [8, 22, 23]. According to a fourth report, an IgA-CA was found to be an early manifestation of MM; this patient had clinical features of CAD, however, with marked symptoms of agglutination and only mild hemolysis [24]. A more recent paper described MM of the IgA type in a patient with CAD, but this report did not include sufficient data to determine whether the patient had one or two cell clones and whether the monoclonal IgA actually was the CA [9]. Römer et al. further characterized an IgA-CA from a subject without clinical CAD and found that this CA was not able to initiate the classical or alternative complement pathway [25]. This is in accordance with a body of evidence showing that erythrocyte-bound IgA does not activate complement [26–28]. Activation of the classical complement pathway is essential for hemolysis in CAD [28, 29].

In conclusion, our patient who developed MM on a background of CAD did not suffer a progress or transformation of CAD-associated LPD but had two distinct disorders. Both CAD and MM responded well to bortezomib-based combination therapy, which is an established treatment option in MM [21]. The combination therapy seemed to induce a deeper remission of CAD as compared with previous rituximab monotherapy, with full normalization of Hb and haptoglobin levels. Therefore, the addition of bortezomib was probably important for this response. A recently published, small prospective trial of bortezomib therapy for CAD found 3 complete responses and 3 partial responses among 19 patients [11]. These response rates may seem low; however, the patients received only one single cycle of monotherapy. It is logical to hope, therefore, that combination therapy and/or extended treatment duration may yield higher response rates. The efficacy of such a combination therapy should be explored in a prospective trial.

In CAD patients with monoclonal gammopathy other than IgMκ, it should be emphasized that the demonstrable monoclonal serum immunoglobulin may not be identical to the CA. Such patients may have two distinct pathological cell clones and two independent diseases. Although IgA-CAs have been convincingly described, CAD of the IgA type does probably not exist.
